# Assessing the accuracy of using diagnostic codes from administrative data to infer antidepressant treatment indications: a validation study

**DOI:** 10.1002/pds.4436

**Published:** 2018-04-23

**Authors:** Jenna Wong, Michal Abrahamowicz, David L. Buckeridge, Robyn Tamblyn

**Affiliations:** ^1^ Department of Epidemiology, Biostatistics, and Occupational Health McGill University Montreal Canada; ^2^ Department of Population Medicine Harvard Medical School and Harvard Pilgrim Health Care Institute Boston MA USA

**Keywords:** administrative data, antidepressant agents, diagnostic codes, indications, pharmacoepidemiology, primary care, validation study

## Abstract

**Purpose:**

To assess the accuracy of using diagnostic codes from administrative data to infer treatment indications for antidepressants prescribed in primary care.

**Methods:**

Validation study of administrative diagnostic codes for 13 plausible indications for antidepressants compared with physician‐documented treatment indications from an indication‐based electronic prescribing system in Quebec, Canada. The analysis included all antidepressant prescriptions written by primary care physicians between January 1, 2003 and December 31, 2012 using the electronic prescribing system. Patient prescribed antidepressants were linked to physician claims and hospitalization data to obtain all diagnoses recorded in the past year.

**Results:**

Diagnostic codes had poor sensitivity for all treatment indications, ranging from a high of only 31.2% (95% CI, 26.8%‐35.9%) for anxiety/stress disorders to as low as 1.3% (95% CI, 0.0%‐5.2%) for sexual dysfunction. Sensitivity was notably worse among older patients and patients with more chronic comorbidities. Physician claims data were a better source of diagnostic codes for antidepressant treatment indications than hospitalization data.

**Conclusions:**

Administrative diagnostic codes are poor proxies for antidepressant treatment indications. Future work should determine whether the use of other variables in administrative data besides diagnostic codes can improve the ability to predict antidepressant treatment indications.

**KEY POINTS**

- Diagnostic codes from administrative health data are often used to infer treatment indications for antidepressant use, but this approach has never been validated against a gold‐standard.
- We found that diagnostic codes in administrative health data had poor accuracy for inferring antidepressant treatment indications when compared with treatment indications documented by primary care physicians at the time of prescribing.
- The findings from this study suggest that use of administrative diagnostic codes to infer antidepressant treatment indications could introduce significant misclassification bias in studies where this approach is used.

## 1 INTRODUCTION

Nearly half of all antidepressants in primary care are prescribed for indications other than depression, including anxiety disorders, insomnia, and pain, among others.^1^ When antidepressants are not prescribed for depression, 2 out of 3 prescriptions are for unapproved (off‐label) indications where in most cases, the drug's use is not supported by strong evidence.^2^ These findings highlight the need for more pharmacovigilance and post‐market evaluations on antidepressant use for indications other than depression.

Employment of information from large administrative databases to evaluate antidepressant use is advantageous because such databases can identify large, population‐based cohorts of antidepressant users, capture many different off‐label uses, and detect rare outcomes or long‐term effects that otherwise might not be observed in clinical trials.^3^ However, administrative databases do not contain information on treatment indications for drugs, which presents a major obstacle for using these data to evaluate antidepressant use for different indications.

In the absence of documented treatment indications, several studies^4^, ^5^, ^6^, ^7^, ^8^ have used administrative diagnostic codes to infer the treatment indication for antidepressant use. However, because this method has never been validated against a reference standard, the potential biases introduced by this approach of inferring antidepressant treatment indications directly from diagnostic codes are unknown. Thus, the objective of this study was to measure the accuracy of using administrative diagnostic codes to infer antidepressant treatment indications, as compared with treatment indications recorded by the prescribing physicians in an indication‐based electronic prescribing system.

## 2 METHODS

### 2.1 Context

This study took place in the Canadian province of Quebec, where all residents are publicly insured for the cost of essential medical care. Over 90% of physicians are reimbursed on a fee‐for‐service basis, with physicians submitting claims to the provincial health insurance agency (the *Régie de l'assurance maladie du Québec* [RAMQ]) for services provided in hospitals or private clinics.^9^ For each claim, physicians can optionally provide a single diagnostic code using the International Classification of Diseases, Ninth Revision (ICD‐9), coding system that represents the main reason for the visit.^10^ Quebec also maintains a hospitalization discharge summary database (MED‐ECHO) containing details of all hospitalizations at acute care institutions in Quebec. Each discharge summary contains a principal diagnosis and up to 15 secondary diagnoses^9^ (up to 25 secondary diagnoses starting in April 2006) recorded by using the ICD‐9 system until April 2006 and the ICD‐10 system thereafter.

### 2.2 Study design

We considered 13 plausible conditions where antidepressants would be used, including various on‐label^11^ and reported off‐label indications^12^, ^13^, ^14^, ^15^ for antidepressants. We conducted a separate validation study for each indication, where the unit of analysis was the prescription.

### 2.3 Data sources and inclusion criteria

The Medical Office of the XXIst Century (MOXXI) is an indication‐based electronic prescribing and drug management system used by consenting primary care physicians at community‐based clinics around 2 major urban centers in Quebec.^16^ The MOXXI electronic prescribing tool requires physicians to document at least 1 treatment indication per prescription using either a drop‐down menu containing on‐label and off‐label indications without distinction, or by typing the indication(s) into a free‐text field. In a previous study,^17^ these physician‐documented treatment indications had excellent sensitivity (98.5%) and high positive predictive value (PPV; 97.0%) when compared with a blinded, post‐hoc physician‐facilitated chart review. Since 2003, 207 physicians (25% of eligible) and over 100 000 patients (26% of all who visited a MOXXI physician) have consented to participate in the MOXXI research program. In general, MOXXI physicians are younger, more technologically proficient, and see fewer patients with less fragmented care than non‐MOXXI physicians, while MOXXI patients are older with more health complexities than non‐MOXXI patients.^18^

This study included all MOXXI prescriptions for any drug approved for depression (see Supporting Information Appendix A) written between January 1, 2003 and December 31, 2012. Patient prescribed antidepressants were linked to the RAMQ and MED‐ECHO databases to obtain all diagnostic codes recorded in physician claims or hospital discharge data over the past 365 days.

This study was approved by the McGill Institutional Review Board.

### 2.4 Study measurements

#### 2.4.1 Antidepressant treatment indications

##### Reference standard

Antidepressant prescriptions were classified as positive for a given indication according to the reference standard (“reference positive”) if the prescriber documented the indication or an ICD subcategory of the indication (eg, “panic attack” under “anxiety disorders”) for the prescription in the MOXXI system. For 1.2% of antidepressant prescriptions that had multiple indications documented, the prescription was classified as reference positive for all the indications.

##### Quebec health administrative databases

Antidepressant prescriptions were classified as positive for a given indication according to administrative data (“test positive”) if the patient had an ICD‐9 code for the indication recorded in either claims (RAMQ) or hospital discharge (MED‐ECHO) data within ±3 days of the prescription date. International Classification of Diseases, Ninth Revision, codes for each indication were identified from code sets used in previous studies^4^, ^19^, ^20^, ^21^ (see Supporting Information Appendix B). For pain, codes for osteoarthritis^22^ and rheumatoid arthritis^23^ were also included because pain is the primary complaint among patients with these conditions.^24^, ^25^ International Classification of Diseases, Tenth Revision, codes recorded in MED‐ECHO from April 2006 onward were translated to their ICD‐9 equivalent using conversion tables.^26^ For 0.6% of antidepressant prescriptions where the patient had diagnostic codes for multiple treatment indications recorded within the time window, the prescription was classified as test positive for all the indications.

#### 2.4.2 Patient characteristics

We determined patients' age and sex by using beneficiary information from RAMQ. We measured patients' level of chronic comorbidity by counting the number of distinct Charlson conditions for which the patient had a corresponding diagnostic code^19^ recorded in administrative data over the past 365 days.

### 2.5 Statistical analysis

For each indication, we conducted a separate validation study to calculate 6 measures of accuracy: sensitivity, specificity, positive predictive value (PPV), negative predictive value (NPV), positive likelihood ratio (LR+), and negative likelihood ratio (LR−) (Table 1). A 2‐stage cluster bootstrap^28^ was used to calculate 95% confidence intervals (CIs) around all accuracy measures corrected for multilevel clustering of prescriptions within patients who in turn were nested within physicians. The reported 95% CIs correspond to the values of the 2.5th and 97.5th percentiles of the distribution of the respective estimates across 1000 bootstrap resamples of the study dataset.

**Table 1:** Measures of accuracy for each antidepressant treatment indication

		Reference Standard (MOXXI)	
		Positive for the Indication	Negative for the Indication
**Administrative data**	**Positive for the indication**	True positive (TP)	False positive (FP)
	**Negative for the indication**	False negative (FN)	True negative (TN)

Measure	Formula	Interpretation using depression as an example
Sensitivity^a^	TP/(TP + FN)	Probability that an antidepressant prescription for depression has a diagnostic code for depression recorded.
Specificity^a^	TN/(TN + FP)	Probability that an antidepressant prescription not for depression does not have a diagnostic code for depression recorded.
Positive predictive value^a^ (PPV)	TP/(TP + FP)	Probability that an antidepressant prescription with a code for depression is truly for depression.
Negative predictive value^a^ (NPV)	TN/(TN + FN)	Probability that an antidepressant prescription without a code for depression is truly not for depression.
Positive likelihood ratio^a^ (LR+)	Sensitivity/(1‐specificity)	How many times more likely it is that a diagnostic code for depression is recorded among prescriptions for depression compared with prescriptions not for depression. Tests with a LR+ of 10 or greater are often considered as having high diagnostic value.^27^
Negative likelihood ratio^b^ (LR−)	(1‐sensitivity)/specificity	How many times more likely it is that a diagnostic code for depression is not recorded among prescriptions for depression compared with prescriptions not for depression. Tests with a LR− of 0.1 or less are often considered as having high diagnostic value.^27^

a Higher values indicate better performance of diagnostic codes for a given indication.

b Lower values indicate better performance of diagnostic codes for a given indication.

#### 2.5.1 Subgroup analyses

For treatment indications with an overall prevalence of >1% according to the reference standard (MOXXI), subgroup analyses were conducted by antidepressant class (selective serotonin re‐uptake inhibitor [SSRI], serotonin‐norepinephrine reuptake inhibitor [SNRI], tricyclic antidepressant [TCA], trazodone, bupropion, or mirtazapine), patient age (<65 versus 65+ years), level of chronic comorbidity (0 versus 1+ Charlson condition), and therapy status (new versus ongoing antidepressant therapy). Prescriptions for new antidepressant therapy were defined as prescriptions where the patient had not been prescribed an antidepressant in MOXXI over the past 365 days.

#### 2.5.2 Sensitivity analyses

We conducted sensitivity analyses to investigate the effect of (a) increasing the lookback window for diagnostic codes (−30, −60, −90, −180, and −365 days) and (b) restricting the source of diagnostic codes to hospital data only, claims data only, or claims from the prescriber only (within a lookback window of 365 days).

To investigate how much of the total variance around each accuracy estimate was due to between‐physician differences in coding practices, the 95% CIs corrected for both within‐patient and within‐physician clustering were compared with 95% CIs corrected for within‐patient clustering only. All analyses were conducted by using SAS software, version 9.4.

## 3 RESULTS

The analysis included a total of 77 700 antidepressant prescriptions written by 164 physicians for 17 606 patients. There were equal numbers of male (n = 82, 50.0%) and female (n = 82, 50%) prescribers; most physicians (n = 150, 91.5%) had received their medical training in Canada or the United States, and 76.6% of physicians (n = 126) had been practicing for at least 15 years. Two thirds of patients were female (n = 11 892, 67.7%), and over the study period, each patient had a median of 3 (interquartile range 1‐6) antidepressant prescriptions. At the time of their earliest antidepressant prescription, most patients were middle aged (median of 53 years, interquartile range 43‐65) and nearly one third (n = 5404, 30.7%) had at least one chronic condition in the Charlson comorbidity index. Among all antidepressant prescriptions, 39.4% (n = 30 596) were initiating new antidepressant therapy. The most commonly prescribed drugs were SSRIs (n = 33 139, 42.7%), followed by SNRIs (n = 18 271, 23.5%), TCAs (n = 8501, 10.9%), trazodone (n = 7216, 9.3%), bupropion (n = 5989, 7.7%), and mirtazapine (n = 4437, 5.7%). Very few prescriptions (<0.2%) were written for monoamine oxidase inhibitors (n = 119), maprotiline (n = 18), or nefazodone (n = 10).

According to the MOXXI indications (reference standard), antidepressants were most commonly prescribed for depression (56.3%), anxiety/stress disorders (22.8%), sleeping disorders (10.0%), and pain (5.7%) (Table 2). In comparison, the proportion of antidepressant prescriptions where the patient had diagnostic codes for these indications (“test positive”) was considerably lower, especially for depression and sleeping disorders (Table 2). Consequently, the sensitivity of administrative diagnostic codes was very poor for all treatment indications, ranging from a high of only 31.2% (95% CI, 26.8%‐35.9%) for anxiety/stress disorders to as low as 1.3% (95% CI, 0.0%‐5.2%) for sexual dysfunction (Table 3). However, the specificity of diagnostic codes was excellent (90%+) for all treatment indications (Table 3).

**Table 2:** Proportion of antidepressant prescriptions for each treatment indication according to MOXXI and Quebec health administrative data

	Number (%) of Antidepressant Prescriptions							
Treatment Indication	MOXXI^a^ [Reference Standard]		Quebec Health Administrative Data^b^		TP	TN	FN	FP
Depressive disorders	43 752	(56.3)	14 465	(18.6)	11 610	31 093	32 142	2 855
Anxiety/stress disorders	17 677	(22.8)	11 606	(14.9)	5 520	53 937	12 ,157	6 086
Sleeping disorders	7 771	(10.0)	720	(0.9)	380	69 589	7 391	340
Pain	4 416	(5.7)	4 090	(5.3)	847	70 041	3 569	3 243
Migraine	1 162	(1.5)	737	(1.0)	259	76 060	903	478
Fibromyalgia	917	(1.2)	796	(1.0)	256	76 243	661	540
Obsessive‐compulsive disorder	840	(1.1)	181	(0.2)	125	76 804	715	56
Vasomotor symptoms of menopause	599	(0.8)	613	(0.8)	48	76 536	551	565
Nicotine dependence	432	(0.6)	108	(0.1)	18	77 178	414	90
Attention deficit/hyperactivity disorder	255	(0.3)	119	(0.2)	23	77 349	232	96
Sexual dysfunction	228	(0.3)	10	(0.0)	3	77 465	225	7
Premenstrual dysphoric disorder	146	(0.2)	26	(0.0)	9	77 537	137	17
Eating disorders	74	(0.1)	31	(0.0)	9	77 604	65	22

Abbreviations: MOXXI, Medical Office of the XXIst Century; TP, true positive; TN, true negative; FN, false negative; FP, false positive.

a Based on physician‐documented treatment indications recorded for antidepressant prescriptions in the MOXXI system. About 1.2% of antidepressant prescriptions were classified as reference positive for multiple treatment indications because more than 1 indication was recorded for the prescription in the MOXXI system.

b Based on diagnostic codes in physician billing and hospitalization discharge summary data that were recorded for patients within ±3 days of the prescription date. About 0.6% of antidepressant prescriptions were classified as test positive for multiple treatment indication because diagnostic codes for more than one treatment indication were recorded.

**Table 3:** Accuracy of diagnostic codes from Quebec health administrative databases for identifying antidepressant treatment indications

Treatment Indication	Prevalence, %	Sensitivity, % (95% CI)	Specificity, % (95% CI)	PPV, % (95% CI)	NPV, % (95% CI)	LR+ (95% CI)	LR− (95% CI)
Depressive disorders	56.3	26.5 (20.7‐32.0)	91.6 (87.6‐94.6)	80.3 (73.7‐85.3)	49.2 (45.3‐53.2)	3.2 (2.3‐4.4)	0.80 (0.75‐0.85)
Anxiety/stress disorders	22.8	31.2 (26.8‐35.9)	89.9 (87.1‐92.3)	47.6 (41.8‐54.3)	81.6 (78.8‐84.0)	3.1 (2.5‐3.9)	0.77 (0.72‐0.81)
Sleeping disorders	10.0	4.9 (3.4‐6.8)	99.5 (99.3‐99.7)	52.8 (46.0‐60.1)	90.4 (88.2‐92.4)	10.1 (7.2‐14.7)	0.96 (0.94‐0.97)
Pain	5.7	19.2 (15.5‐23.0)	95.6 (94.8‐96.3)	20.7 (16.4‐25.9)	95.2 (94.2‐95.9)	4.3 (3.5‐5.4)	0.85 (0.81‐0.88)
Migraine	1.5	22.3 (17.0‐29.1)	99.4 (99.2‐99.5)	35.1 (26.2‐45.2)	98.8 (98.4‐99.2)	35.7 (27.0‐49.2)	0.78 (0.71‐0.84)
Fibromyalgia	1.2	27.9 (18.8‐38.8)	99.3 (99.0‐99.5)	32.2 (23.9‐40.2)	99.1 (98.9‐99.4)	39.7 (28.1‐55.5)	0.73 (0.62‐0.82)
Obsessive‐compulsive disorder	1.1	14.9 (7.5‐23.4)	99.9 (99.9‐100.0)	69.1 (51.7‐83.3)	99.1 (98.8‐99.3)	203.8 (103.1‐452.2)	0.85 (0.77‐0.93)
Vasomotor symptoms of menopause	0.8	8.0 (3.8‐13.3)	99.3 (98.9‐99.5)	7.8 (3.5‐14.3)	99.3 (99.0‐99.5)	10.9 (5.1‐20.7)	0.93 (0.87‐0.97)
Nicotine dependence	0.6	4.2 (0.7‐9.4)	99.9 (99.8‐99.9)	16.7 (4.2‐29.3)	99.5 (99.2‐99.7)	35.9 (8.2‐73.8)	0.96 (0.91‐0.99)
Attention deficit/hyperactivity disorder	0.3	9.0 (2.1‐17.3)	99.9 (99.8‐99.9)	19.3 (5.3‐37.1)	99.7 (99.5‐99.8)	72.7 (20.3‐178.8)	0.91 (0.83‐0.98)
Sexual dysfunction	0.3	1.3 (0.0‐5.2)	100.0 (100.0‐100.0)	30.0 (0.0‐88.9)	99.7 (99.5‐99.9)	146.2 (0.0‐1337.5)	0.99 (0.95‐1.00)
Pre‐menstrual dysphoric disorder	0.2	6.2 (0.0‐15.4)	100.0 (99.9‐100.0)	34.6 (0.0‐71.4)	99.8 (99.7‐99.9)	280.2 (0.0‐1434.5)	0.94 (0.84‐1.00)
Eating disorders	0.1	12.2 (0.0‐32.8)	100.0 (99.9‐100.0)	29.0 (0.0‐66.7)	99.9 (99.8‐100.0)	434.4 (0.0‐2111.6)	0.88 (0.67‐1.00)

Abbreviations: PPV, positive predictive value; NPV, negative predictive value; LR+, positive likelihood ratio; LR, negative likelihood ratio.

The predictive value of having an administrative diagnostic code for a given indication recorded varied between indications. When a diagnostic code for a given indication was recorded, the probability that the antidepressant was truly prescribed for the corresponding indication (ie, according to MOXXI) was high for depression (PPV of 80.3%; 95% CI, 73.7%‐85.3%), moderate for obsessive‐compulsive disorder (OCD) (69.1%; 95% CI, 51.7%‐83.3%), and low (~50% or less) for the remaining indications (Table 3). The high PPV of depression codes was mostly attributable to the high prevalence (or baseline probability) of depression (56.3%), whereas OCD codes had a PPV of 69.1% despite the indication having a very low prevalence of only 1.1%. The contrast in predictive value of diagnostic codes for these indications was better displayed by the LR+ because it was not influenced by the prevalence of these indications. Diagnostic codes for depression had an LR+ of only 3.2 (95% CI, 2.3‐4.4) compared with 203.8 (95% CI, 103.1‐452.2) for OCD codes, suggesting that OCD codes were much more informative than depression codes for ruling in the corresponding indication.

Similarly, conclusions about the predictive value of *not* having a diagnostic code recorded for a given indication differed depending on whether the NPV or LR− was used as the performance statistic. When a diagnostic code for a given indication was not recorded, the probability that the antidepressant was not prescribed for the corresponding indication in MOXXI was low for depression (NPV of 49.2%; 95% CI, 45.3%‐53.2%) but fairly high for anxiety/stress disorders (81.6%; 95% CI, 78.8%‐84.0%) and high for sleeping disorders (90.4%; 95% CI, 88.2%‐92.4%). For the remaining indications, the NPV was very high (>95%) because of the low prevalence of these indications (Table 3). In contrast, the LR− estimates were close to 1.0 for all indications, suggesting that the absence of a diagnostic code for any plausible indication did not improve the ability to rule out the corresponding indication.

### 3.1 Subgroup analyses

For all indications, there was considerable heterogeneity in the PPV and NPV estimates across different classes of antidepressants (Table 4). Diagnostic codes usually had better PPV and poorer NPV for antidepressants with a higher prevalence of the indication. However, there were 2 exceptions to this trend. For fibromyalgia, the baseline probability of this indication was similar for SNRIs and TCAs (3.1% versus 3.4%) but the PPV for SNRIs (62.7%; 95% CI, 47.5%‐76.0%) was much higher than for TCAs (32.0%; 95% CI, 16.9%‐45.9%). Similarly, the baseline probability of OCD was low for both SSRIs and SNRIs (2.0% versus 0.7%), yet the PPV for SSRIs (81.0%; 95% CI, 62.5%‐94.4%) was much higher than for SNRIs (38.1%; 95% CI, 0.0%‐77.8%). Unlike the PPV and NPV, the LR estimates were less heterogeneous between different antidepressant classes and did not depend on the prevalence of the indication (see Supporting Information Appendix C).

**Table 4:** Positive predictive value (PPV) and negative predictive value (NPV) of administrative diagnostic codes for the 7 most common treatment indications, by antidepressant class

Treatment Indication, by Antidepressant Class^a^	Prevalence, %	PPV, % (95% CI)		NPV, % (95% CI)	
Depressive disorders					
SSRI	61.9	86.1	(82.2‐89.4)	43.4	(38.6‐48.7)
SNRI	67.1	88.3	(84.5‐91.4)	39.3	(34.0‐44.5)
TCA	14.7	27.3	(13.2‐51.9)	86.4	(82.6‐89.8)
Trazodone	10.4	20.3	(9.6‐36.0)	91.3	(85.9‐95.5)
Bupropion	84.1	93.1	(89.0‐97.0)	19.5	(14.0‐25.1)
Mirtazapine	86.9	96.5	(93.5‐98.6)	15.1	(10.2‐21.0)
Anxiety/stress disorders					
SSRI	36.0	63.2	(56.0‐70.4)	70.2	(65.3‐74.4)
SNRI	24.1	51.3	(41.9‐62.0)	80.8	(77.3‐84.0)
TCA	3.2	7.1	(2.9‐14.0)	97.1	(95.6‐98.3)
Trazodone	7.8	8.9	(4.4‐14.3)	92.4	(89.0‐95.0)
Bupropion	0.3	1.2	(0.0‐3.2)	99.8	(99.6‐100.0)
Mirtazapine	10.3	15.3	(9.0‐26.6)	90.5	(84.8‐94.8)
Sleeping disorders					
SSRI	0.0	0.0	(0.0‐0.0)	100.0	(99.9‐100.0)
SNRI	0.0	1.5	(0.0‐6.8)	100.0	(100.0‐100.0)
TCA	20.0	67.5	(44.7‐84.3)	80.5	(72.2‐87.5)
Trazodone	82.0	95.8	(90.8‐98.8)	18.6	(13.1‐25.4)
Bupropion	0.0	0.0	(0.0‐0.0)	100.0	(100.0‐100.0)
Mirtazapine	3.2	21.0	(3.5‐31.5)	97.4	(95.0‐99.3)
Pain					
SSRI	0.1	0.4	(0.0‐1.1)	100.0	(99.9‐100.0)
SNRI	3.1	15.7	(9.3‐23.4)	97.6	(96.7‐98.3)
TCA	42.8	72.0	(62.9‐79.6)	60.8	(53.2‐67.5)
Trazodone	1.6	4.4	(0.9‐9.6)	98.6	(97.4‐99.4)
Bupropion	1.3	5.1	(0.3‐11.9)	98.8	(97.9‐99.5)
Mirtazapine	0.0	0.0	(0.0‐0.0)	100.0	(100.0‐100.0)
Migraine					
SSRI	0.0	2.2	(0.0‐7.2)	100.0	(99.9‐100.0)
SNRI	0.0	0.0	(0.0‐0.0)	100.0	(99.9‐100.0)
TCA	13.5	71.6	(61.5‐80.5)	89.0	(85.1‐92.4)
Trazodone	0.0	0.0	(0.0‐0.0)	100.0	(100.0‐100.0)
Bupropion	0.0	0.0	(0.0‐0.0)	100.0	(100.0‐100.0)
Mirtazapine	0.0	0.0	(0.0‐0.0)	100.0	(100.0‐100.0)
Fibromyalgia					
SSRI	0.1	7.9	(0.0‐20.5)	99.9	(99.7‐100.0)
SNRI	3.1	62.7	(47.5‐76.0)	97.7	(96.8‐98.5)
TCA	3.4	32.0	(16.9‐45.9)	97.5	(96.3‐98.6)
Trazodone	0.0	1.4	(0.0‐7.3)	100.0	(99.9‐100.0)
Bupropion	0.3	30.0	(0.0‐64.3)	99.9	(99.6‐100.0)
Mirtazapine	0.0	0.0	(0.0‐0.0)	100.0	(100.0‐100.0)
Obsessive‐compulsive disorder					
SSRI	2.0	81.0	(62.5‐94.4)	98.3	(97.8‐98.8)
SNRI	0.7	38.1	(0.0‐77.8)	99.3	(98.7‐99.7)
TCA	0.4	85.7	(0.0‐100.0)	99.6	(99.2‐99.9)
Trazodone	0.0	0.0	(0.0‐0.0)	100.0	(100.0‐100.0)
Bupropion	0.1	0.0	(0.0‐0.0)	100.0	(99.8‐100.0)
Mirtazapine	0.0	N/A^b^		100.0	(100.0‐100.0)

Abbreviations: SSRI, selective serotonin reuptake inhibitor; SNRI, serotonin‐norepinephrine reuptake inhibitor; TCA, tricyclic antidepressant.

a SSRIs include citalopram, paroxetine, sertraline, escitalopram, fluoxetine, and fluvoxamine. SNRIs include venlafaxine, duloxetine, and desvenlafaxine. TCAs include amitriptyline, doxepin, trimipramine, nortriptyline, imipramine, clomipramine, and desipramine. Results are not shown for the monoamine oxidase inhibitors (moclobemide, phenelzine, and tranylcypromine), maprotiline, or nefazodone due to small numbers of prescriptions for each of these drugs.

b Could not be calculated because of a zero denominator because no prescriptions for mirtazapine had a diagnostic code for obsessive‐compulsive disorder recorded within ±3 days of the prescription date.

When prescriptions were stratified by patients' level of chronic comorbidity and age, diagnostic codes for all indications had noticeably poorer sensitivity among patients with at least 1 chronic condition in the Charlson index and patients 65+ years old, especially for depression and anxiety/stress disorders (Tables 5 and 6). Although the stratum‐specific estimates for sicker and older patients were similar, they were not entirely dependent on each other because these 2 patient characteristics were only weakly positively correlated (Pearson's *r* = 0.285). Among prescriptions for new versus ongoing antidepressant therapy, the sensitivity and PPV of diagnostic codes was better among prescriptions for new antidepressant therapy for all indications except depression and fibromyalgia (Table 7).

**Table 5:** Sensitivity and specificity of diagnostic codes for the 7 most common treatment indications, by level of patient chronic comorbidity

Treatment Indication	Sensitivity, % (95% CI)		Specificity, % (95% CI)	
	0 Charlson Conditions	1+ Charlson Conditions	0 Charlson Conditions	1+ Charlson Conditions
Depressive disorders	31.2 (24.3‐37.8)	16.7 (12.8‐20.3)	90.2 (85.2‐93.9)	94.7 (92.5‐96.6)
Anxiety/stress disorders	35.8 (30.4‐41.0)	19.5 (16.0‐23.5)	88.3 (85.3‐91.3)	93.0 (91.2‐94.6)
Sleeping disorders	5.0 (3.3‐7.1)	4.7 (2.9‐6.4)	99.5 (99.3‐99.7)	99.5 (99.2‐99.7)
Pain	21.0 (16.6‐25.4)	16.3 (11.3‐21.0)	95.9 (95.2‐96.6)	94.8 (93.8‐95.8)
Migraine	25.3 (18.9‐33.3)	12.6 (7.0‐21.2)	99.3 (99.0‐99.5)	99.6 (99.4‐99.7)
Fibromyalgia	33.0 (21.9‐43.2)	16.9 (6.6‐29.5)	99.2 (98.9‐99.5)	99.4 (99.2‐99.7)
Obsessive‐compulsive disorder	16.4 (8.5‐26.2)	8.5 (0.5‐21.5)	99.9 (99.8‐100.0)	100.0 (99.9‐100.0)

**Table 6:** Sensitivity and specificity of diagnostic codes for the 7 most common treatment indications, by patient age

Treatment Indication	Sensitivity, % (95% CI)		Specificity, % (95% CI)	
	<65 Years	65+ Years	<65 Years	65+ Years
Depressive disorders	29.9 (23.6‐36.5)	16.0 (12.8‐20.0)	89.9 (85.3‐93.5)	96.1 (94.1‐97.5)
Anxiety/stress disorders	35.7 (30.7‐40.4)	18.4 (14.2‐23.3)	88.6 (85.6‐91.4)	93.7 (92.0‐95.3)
Sleeping disorders	5.1 (3.3‐7.3)	4.5 (2.9‐6.5)	99.5 (99.2‐99.7)	99.6 (99.4‐99.8)
Pain	20.7 (16.4‐25.0)	16.0 (11.0‐21.5)	95.9 (95.2‐96.7)	94.4 (93.5‐95.4)
Migraine	24.1 (17.6‐31.7)	13.0 (4.8‐24.0)	99.3 (99.0‐99.5)	99.7 (99.5‐99.8)
Fibromyalgia	29.6 (18.8‐40.5)	20.2 (6.1‐36.0)	99.2 (98.9‐99.5)	99.6 (99.4‐99.8)
Obsessive‐compulsive disorder	17.0 (9.0‐27.4)	2.4 (0.0‐9.9)	99.9 (99.8‐100.0)	100.0 (99.9100.0)

**Table 7:** Sensitivity and positive predictive value (PPV) of diagnostic codes for the 7 most common treatment indications, by antidepressant therapy status

Treatment Indication	Sensitivity, % (95% CI)		PPV, % (95% CI)	
	New Therapy^a^	Ongoing Therapy	New Therapy^a^	Ongoing Therapy
Depressive disorders	26.1 (21.7‐29.9)	26.8 (20.2‐33.3)	80.8 (72.6‐87.0)	80.0 (74.0‐84.8)
Anxiety/stress disorders	33.7 (28.5‐38.3)	29.5 (24.8‐34.3)	52.4 (45.5‐59.7)	44.3 (38.0‐52.0)
Sleeping disorders	6.6 (4.5‐9.0)	3.6 (2.1‐5.3)	61.9 (52.7‐71.5)	43.8 (34.4‐53.5)
Pain	20.4 (16.2‐24.3)	18.2 (13.4‐23.1)	26.1 (20.6‐31.4)	17.4 (12.6‐23.0)
Migraine	28.0 (21.5‐34.7)	16.3 (10.1‐25.9)	48.0 (37.0‐58.8)	23.7 (15.0‐34.9)
Fibromyalgia	26.3 (16.6‐36.3)	28.7 (18.1‐40.8)	32.4 (22.2‐42.7)	32.0 (22.7‐42.3)
Obsessive‐compulsive disorder	17.0 (7.5‐28.1)	13.4 (5.3‐23.3)	71.6 (51.9‐87.2)	67.0 (44.3‐85.4)

a Defined as prescriptions where the patient had not been prescribed an antidepressant in the Medical Office of the XXIst Century system over the past 365 days.

### 3.2 Sensitivity analyses

**Figure 1:**
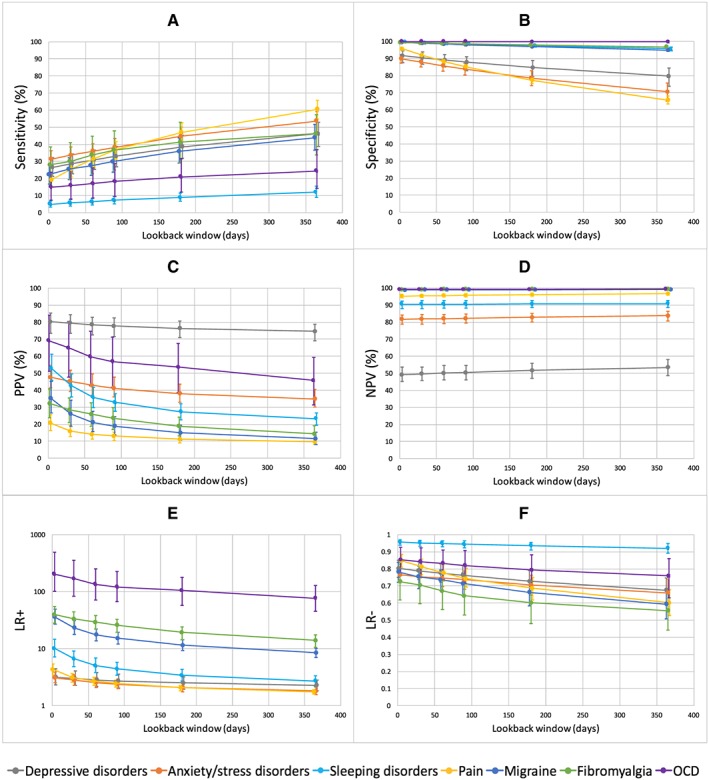
Effect of increasing the lookback window for administrative diagnostic codes. The figure shows the classification parameter estimates for the 7 most common treatment indications based on administrative diagnostic codes recorded in the past 3, 30, 60, 90, 180, and 365 days. Abbreviations: PPV, positive predictive value; NPV, negative predictive value; LR+, positive predictive value; LR−, negative predictive value [Colour figure can be viewed at http://wileyonlinelibrary.com]

As expected, using a longer lookback window for diagnostic codes increased sensitivity and decreased specificity for all indications, especially pain (Figure 1A,B). However, even with a lookback window of −365 days, sensitivity remained low at ≤60% for all indications. Increasing the length of the lookback window also caused the PPV and LR+ to deteriorate for all indications (Figure 1C,E).

**Figure 2:**
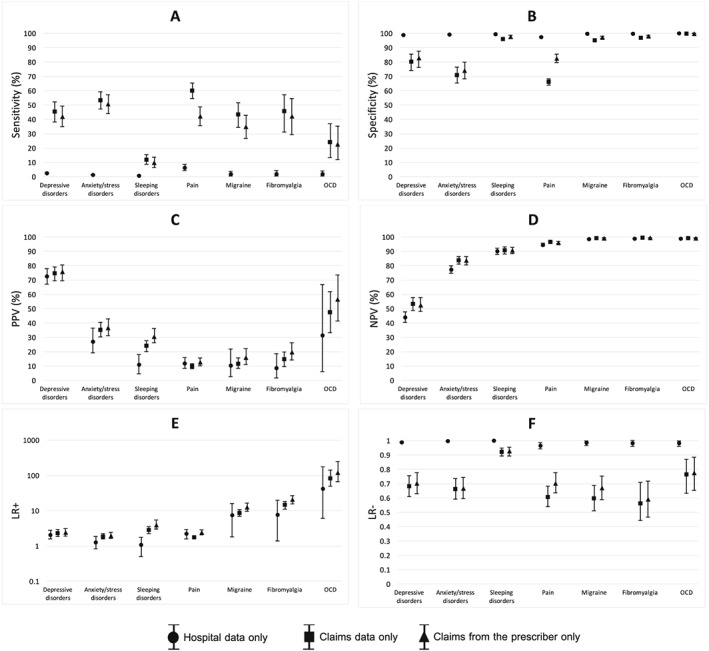
Effect of restricting diagnostic codes to different sources of administrative data. The figure shows the classification parameter estimates for the 7 most common treatment indications based on diagnostic codes recorded within the past 365 days when restricted to diagnostic codes from either hospital discharge data, billings from all physicians, or billings from the prescribing physician only. Abbreviations: PPV, positive predictive value; NPV, negative predictive value; LR+, positive predictive value; LR−, negative predictive value

Compared with the performance of diagnostic codes from claims data in the past 365 days, diagnostic codes from hospital data in the past 365 days had drastically lower sensitivity for all indications (Figure 2A). However, when diagnostic codes from claims data in the past 365 days were restricted from all physicians to those from the prescriber only, the sensitivity of diagnostic codes was notably lower for pain only (Figure 2A). Diagnostic codes recorded by the prescriber also had slightly higher (better) PPV and LR+ than diagnostic codes recorded by all physicians (Figure 2C,E).

**Figure 3:**
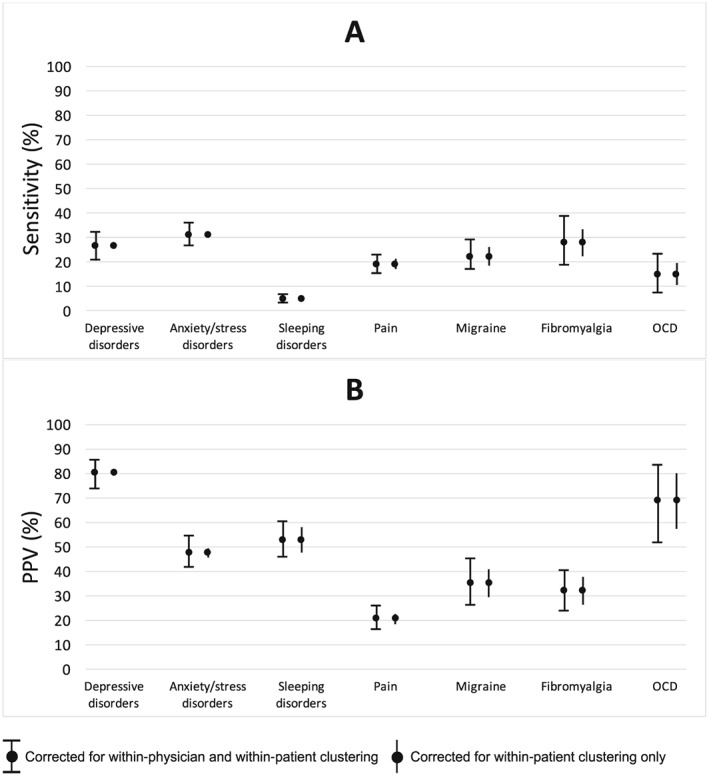
Variance of the sensitivity and positive predictive value (PPV) estimates when corrected for both within‐physician and within‐patient clustering versus within‐patient clustering only. The figure shows the width of the 95% CIs around the sensitivity (panel A) and PPV estimates (panel B) when a 2‐stage cluster bootstrap^28^ was used to correct for clustering of prescriptions within patients who in turn were nested within physicians (capped vertical bars) versus when a 1‐stage cluster bootstrap was used to correct for only clustering of prescriptions within patients (uncapped vertical bars). The upper and lower bounds of the 95% CI correspond to the values of the 2.5th and 97.5th percentiles of the distribution of the respective estimates across 1000 bootstrap re‐samples. Results are shown for the 7 most common treatment indications based on diagnostic codes recorded in administrative data within ±3 days of the prescription date

Finally, for all indications except sleeping disorders, the 95% cluster bootstrap‐based CIs^28^ around the sensitivity and PPV estimates were noticeably wider when they accounted for both within‐physician and within‐patient clustering than when they accounted for within‐patient clustering only, suggesting that within‐physician differences exist in the quality of diagnostic coding for these indications, especially depression (Figure 3).

## 4 DISCUSSION

In this study, we estimated the accuracy with which diagnostic codes in Quebec health administrative records reflected indications for antidepressant therapy in primary care. We found that diagnostic codes for a given indication identified only a small proportion of antidepressant prescriptions for the corresponding indication. Moreover, we found that the absence of a diagnostic code for a given indication did not provide much additional value for ruling out the indication.

The findings from this validation study have important implications for epidemiological studies using administrative diagnostic codes to infer antidepressant treatment indications. Studies aimed at monitoring rates of antidepressant use for off‐label indications will significantly overestimate the true off‐label prescribing rate since a large proportion of truly on‐label antidepressant prescriptions will not have a corresponding diagnostic code for the indication. Our findings also suggest that in safety studies of off‐label antidepressant use, the use of administrative diagnostic codes to infer treatment indications could misclassify a significant proportion of on‐label users as off‐label users, thus possibly diluting or even concealing adverse drug events among off‐label users. For example, in the case of mirtazapine (approved in Canada for depression only), we found that diagnostic codes for depression had an NPV of only 15.1% (95% CI, 10.2%‐21.0%), suggesting that 84.9% (95% CI, 79.0%‐89.8%) of supposedly off‐label mirtazapine users could in fact be on‐label users (since they do not have a diagnostic code for depression but have been prescribed trazodone to treat depression). This example illustrates a scenario where the accuracy estimates from this study could be useful for informing bias analyses in studies where administrative diagnostic codes have been used to infer antidepressant treatment indications.

Our study highlights the importance of considering disease prevalence when interpreting and comparing the PPV and NPV of diagnostic tests. We found that diagnostic codes for a given indication generally had better PPV and worse NPV among antidepressants with a high rather than low prevalence of the indication. Thus, in validation studies, it is important not only to report the disease prevalence in the study population but also to stratify the analysis by factors that are expected to affect disease prevalence in the study population. Furthermore, we found that the LR estimates were unaffected by the different prevalences of the indications, suggesting that it may be useful to consider these statistical measures alongside the PPV and NPV when assessing the predictive properties of a diagnostic test.

Two main factors help explain the poor accuracy of diagnostic codes for antidepressant treatment indications in Quebec administrative data. First, physicians have little incentive to accurately record diagnostic codes when completing medical claims since they are not required to submit diagnostic codes. Second, since only one diagnosis can be recorded per claim, this limitation reduces the likelihood that a code for the antidepressant treatment indication will be recorded, especially among patients with multiple morbidities. Indeed, we found that administrative diagnostic codes had lower sensitivity among patients with higher levels of chronic comorbidity.

Our finding that the sensitivity of pain codes was much lower when restricted to claims from the prescriber compared with claims from all physicians suggests that patients who are prescribed antidepressants for pain are likely to seek treatment from multiple physicians. However, the fact that we did not observe this finding for other indications suggests that primary care physicians may often provide most of the care for these conditions.

This study has several limitations. First, although the treatment indications we validated in this study accounted for 99.5% of antidepressant prescriptions in the MOXXI system, we did not validate the indications for the remaining 0.5% of prescriptions (eg, fatigue, bipolar disorder, obesity, Crohn's disease, irritable bowel syndrome, cocaine dependence, and alcoholism) because they were so rare. Second, the external generalizability of our findings depends on the extent to which diagnostic coding practices are similar between MOXXI physicians and physicians in other settings. MOXXI physicians operate within a publicly funded health care system, whereas in other countries like the United States where health care is heavily privatized, physicians have been known to compromise their coding practices for depression due to concerns over obtaining reimbursement or jeopardizing patients' future ability to obtain health insurance.^29^ Another limitation of our study is that we could not determine how often MOXXI physicians recorded only one indication for the prescription when there were truly multiple indications. If certain indications were often omitted, then we may have overestimated the NPV and underestimated the PPV of diagnostic codes for these indications. Finally, in our main analysis, we used a short lookback window of 3 days for diagnostic codes because we knew when the index prescription was written. For researchers using dispensing data where the date of the index visit is unknown, a longer lookback window may be necessary to capture the index visit.

In conclusion, the findings from this study suggest that diagnostic codes from administrative data are poor proxies for antidepressant treatment indications and should not be used alone to infer treatment indications. Future studies should determine whether diagnostic codes can be combined with other information from administrative health databases to improve the ability to predict antidepressant treatment indications.

## ETHICS STATEMENT

Ethics approval was obtained for this study from the McGill Institutional Review Board. All MOXXI physicians and patients have consented to have their information used for research purposes.

## FUNDING

This work was supported by the Vanier Canada Graduate Scholarship (Canadian Institutes of Health Research), the Max E. Binz fellowship from the Faculty of Medicine at McGill University, a graduate student fellowship from the Research Institute of the McGill University Health Centre, and grant IOP‐112675 from the Canadian Institutes of Health Research. The funding agency did not have any role in study design; in the collection, analysis, and interpretation of the data; in the writing of the report; and in the decision to submit the article for publication.

## Supporting information


**Appendix A.** Names and ATC codes of drugs included in the analysis
**APPENDIX B.** ICD–9 codes for antidepressant treatment indication
**APPENDIX C.** Positive likelihood ratio (LR+) and negative likelihood ratio (LR−) of administrative diagnostic codes for the seven most common treatment indications, by antidepressant class
