# Supplementary material for: Assessing the accuracy of using diagnostic codes from administrative data to infer antidepressant treatment indications: a validation study
Source: Pharmacoepidemiol Drug Saf. 2018 Apr 23;27(10):1101–11. doi: 10.1002/pds.4436 (PMC6220980; doi:10.1002/pds.4436)
Supplement: Supplementary file 1 — Appendix A. Names and ATC codes of drugs included in the analysis APPENDIX B. ICD–9 codes for antidepressant treatment indication APPENDIX C. Positive likelihood ratio (LR+) and negative likelihood ratio (LR−) of administrative diagnostic codes for the seven most common treatment indications, by antidepressant class [file PDS-27-1101-s001.docx]

**Appendix A. Names and ATC codes of drugs included in the analysis**

| **Pharmacological class** | **Drug name** | **ATC code** |
| --- | --- | --- |
| Selective serotonin reuptake inhibitor | Citalopram | N06AB04 |
|  | Escitalopram | N06AB10 |
|  | Fluvoxamine | N06AB08 |
|  | Fluoxetine | N06AB03 |
|  | Paroxetine | N06AB05 |
|  | Sertraline | N06AB06 |
| Serotonin-norepinephrine reuptake inhibitor | Desvenlafaxine | N06AX23 |
|  | Duloxetine | N06AX21 |
|  | Venlafaxine | N06AX16 |
| Tricyclic antidepressant | Amitriptyline | N06AA09 |
|  | Clomipramine | N06AA04 |
|  | Desipramine | N06AA01 |
|  | Doxepin | N06AA12 |
|  | Imipramine | N06AA02 |
|  | Nortriptyline | N06AA10 |
|  | Trimipramine | N06AA06 |
| Monoamine oxidase inhibitors | Moclobemide | N06AG02 |
|  | Phenelzine | N06AF03 |
|  | Tranylcypromine | N06AF04 |
| Other | Bupropion | N06AX12 |
|  | Maprotiline | N06AA21 |
|  | Mirtazapine | N06AX11 |
|  | Nefazodone | N06AX06 |
|  | Trazodone | N06AX05 |

**APPENDIX B. ICD-9 codes for antidepressant treatment indications**

| **Treatment indication** | **ICD-9 code** | **Code description** | **No. prescriptions with diagnostic code recorded** | |
| --- | --- | --- | --- | --- |
|  |  |  | **Within -3 to +3 days** | **Within -365 to +3 days** |
| Depressive disorders | 296.2 | Major depressive disorder, single episode | 250 | 965 |
|  | 296.3 | Major depressive disorder, recurrent episode | 169 | 576 |
|  | 296.9 | Other and unspecified episodic mood disorder | 24 | 462 |
|  | 300.4 | Dysthymic disorder | 3467 | 7886 |
|  | 301.1 | Affective personality disorder (includes chronic depressive personality disorder) | 79 | 258 |
|  | 309.0 | Adjustment disorder with depressed mood | 2170 | 5475 |
|  | 309.1 | Prolonged depressive reaction | 60 | 115 |
|  | 311.x | Depressive disorder, not elsewhere classified | 8317 | 16265 |
| Anxiety/stress disorders | 300.0 | Anxiety states | 10779 | 25933 |
|  | 300.2 | Phobic disorders | 445 | 945 |
|  | 308.x | Acute reaction to stress | 130 | 477 |
|  | 309.2 | Adjustment reaction with predominant disturbance of other emotions | 57 | 379 |
|  | 309.8 | Other specified adjustment reactions (includes posttraumatic stress disorder) | 203 | 434 |
| Sleeping disorders | 307.4 | Specific disorders of sleep of nonorganic origin | 21 | 123 |
|  | 780.5 | Sleep disturbances | 699 | 3914 |

**APPENDIX B. (continued) ICD-9 codes for antidepressant treatment indications**

| **Treatment indication** | **ICD-9 code** | **Code description** | **No. prescriptions with diagnostic code recorded** | |
| --- | --- | --- | --- | --- |
|  |  |  | **Within -3 to +3 days** | **Within -365 to +3 days** |
| Pain | 053.1 | Herpes zoster with other nervous system complications | 11 | 31 |
|  | 250.6 | Diabetes with neurological manifestations | 5 | 125 |
|  | 307.8 | Pain disorders related to psychological factors | 24 | 202 |
|  | 337.2 | Reflex sympathetic dystrophy | 0 | 0 |
|  | 338.x | Pain, not elsewhere classified | 0 | 0 |
|  | 350.1 | Trigeminal neuralgia | 3 | 55 |
|  | 350.2 | Atypical face pain | 14 | 53 |
|  | 352.1 | Glossopharyngeal neuralgia | 0 | 0 |
|  | 353.X | Nerve root and plexus disorders | 7 | 98 |
|  | 354.x | Mononeuritis of upper limb and mononeuritis multiplex | 80 | 1393 |
|  | 355.x | Mononeuritis of lower limb | 53 | 603 |
|  | 357.2 | Polyneuropathy in diabetes | 34 | 220 |
|  | 714.x | Rheumatoid arthritis and other inflammatory polyarthropathies | 250 | 1113 |
|  | 715.x | Osteoarthrosis and allied disorders | 881 | 6168 |
|  | 719.4 | Pain in joint | 36 | 452 |
|  | 721.x | Spondylosis and allied disorders | 53 | 519 |
|  | 722.x | Intervertebral disc disorders | 169 | 1488 |
|  | 723.x | Other disorders of cervical region | 207 | 2039 |
|  | 724.x | Other and unspecified disorders of back | 1069 | 7593 |
|  | 729.2 | Neuralgia, neuritis, and radiculitis, unspecified | 86 | 943 |
|  | 729.5 | Pain in limb | 324 | 4735 |
|  | 737.x | Curvature of spine | 3 | 44 |

**APPENDIX B. (continued) ICD-9 codes for antidepressant treatment indications**

| **Treatment indication** | **ICD-9 code** | **Code description** | **No. prescriptions with diagnostic code recorded** | |
| --- | --- | --- | --- | --- |
|  |  |  | **Within -3 to +3 days** | **Within -365 to +3 days** |
| Pain (continued) | 786.5 | Chest pain | 329 | 5943 |
|  | 789.0 | Abdominal pain | 538 | 7923 |
| Migraine | 346.x | Migraine | 492 | 2230 |
|  | 784.0 | Headache | 248 | 2714 |
| Fibromyalgia | 729.1 | Myalgia and myositis, unspecified | 796 | 2977 |
| Obsessive-compulsive disorder | 300.3 | Obsessive-compulsive disorder | 181 | 449 |
| Vasomotor symptoms of menopause | 627.2 | Symptomatic menopausal or female climacteric states | 613 | 3393 |
| Nicotine dependence | 305.1 | Tobacco use disorder | 108 | 574 |
| Attention deficit/hyperactivity disorder | 314.x | Hyperkinetic syndrome of childhood | 119 | 460 |
| Sexual dysfunction | 302.7 | Psychosexual dysfunction | 10 | 79 |
|  | 607.8 | Other specified disorders of penis | 0 | 27 |
| Pre-menstrual dysphoric disorder | 625.4 | Premenstrual tension syndromes | 26 | 107 |
| Eating disorders | 307.1 | Anorexia nervosa | 5 | 44 |
|  | 307.5 | Other and unspecified disorders of eating | 8 | 56 |
|  | 783.0 | Anorexia | 15 | 77 |
|  | 783.3 | Feeding difficulties and mismanagement | 4 | 13 |

**APPENDIX C. Positive likelihood ratio (LR+) and negative likelihood ratio (LR-) of administrative diagnostic codes for the seven most common treatment indications, by antidepressant class**

| **Treatment indication, by antidepressant class^†^** | **Prevalence (%)** | **LR+ (95% CI)** | | **LR- (95% CI)** | |
| --- | --- | --- | --- | --- | --- |
| **Depressive disorders** |  |  |  |  |  |
| SSRI | 61.9 | 3.82 | (2.93-5.13) | 0.80 | (0.75-0.86) |
| SNRI | 67.1 | 3.69 | (2.67-5.44) | 0.76 | (0.70-0.82) |
| TCA | 14.7 | 2.18 | (1.04-5.41) | 0.91 | (0.83-0.99) |
| Trazodone | 10.4 | 2.18 | (1.19-3.73) | 0.82 | (0.67-0.96) |
| Bupropion | 84.1 | 2.56 | (1.62-5.58) | 0.78 | (0.69-0.88) |
| Mirtazapine | 86.9 | 4.13 | (2.11-10.43) | 0.85 | (0.79-0.91) |
| **Anxiety/stress disorders** |  |  |  |  |  |
| SSRI | 36.0 | 3.06 | (2.30-4.07) | 0.76 | (0.70-0.81) |
| SNRI | 24.1 | 3.32 | (2.46-4.78) | 0.75 | (0.69-0.80) |
| TCA | 3.2 | 2.32 | (1.00-4.50) | 0.91 | (0.79-1.00) |
| Trazodone | 7.8 | 1.15 | (0.58-1.91) | 0.98 | (0.89-1.05) |
| Bupropion | 0.3 | 4.29 | (0.00-8.75) | 0.60 | (0.10-1.11) |
| Mirtazapine | 10.3 | 1.57 | (0.97-2.52) | 0.91 | (0.78-1.00) |
| **Sleeping disorders** |  |  |  |  |  |
| SSRI | 0.0 | 0.00 | (0.00-0.00) | 1.00 | (1.00-1.00) |
| SNRI | 0.0 | 136.24 | (0.00-285.71) | 0.50 | (0.00-1.00) |
| TCA | 20.0 | 8.32 | (3.51-23.64) | 0.97 | (0.95-0.99) |
| Trazodone | 82.0 | 4.94 | (2.04-19.26) | 0.96 | (0.95-0.98) |
| Bupropion | 0.0 | N/A^‡^ |  | N/A^‡^ |  |
| Mirtazapine | 3.2 | 8.03 | (3.24-18.62) | 0.81 | (0.71-0.95) |

Abbreviations: SSRI = selective serotonin reuptake inhibitor; SNRI = serotonin-norepinephrine reuptake inhibitor; TCA = tricyclic antidepressant

^†^SSRIs include citalopram, paroxetine, sertraline, escitalopram, fluoxetine, and fluvoxamine. SNRIs include venlafaxine, duloxetine, and desvenlafaxine. TCAs include amitriptyline, doxepin, trimipramine, nortriptyline, imipramine, clomipramine, and desipramine. Results are not shown for monoamine oxidase inhibitors, maprotiline, or nefazodone due to small numbers of prescriptions for each of these drugs.

^‡^Because no prescriptions for the antidepressant were written for the indication, the positive and negative likelihood ratio could not be calculated due to a zero denominator for the true positive rate (sensitivity) and false positive rate (1-sensitivity), respectively.

**APPENDIX C. (continued) Positive likelihood ratio (LR+) and negative likelihood ratio (LR-) of administrative diagnostic codes for the seven most common treatment indications, by antidepressant class**

| **Treatment indication, by antidepressant class^†^** | **Prevalence (%)** | **LR+ (95% CI)** | | **LR- (95% CI)** | |
| --- | --- | --- | --- | --- | --- |
| **Pain** |  |  |  |  |  |
| SSRI | 0.1 | 6.06 | (0.00-12.93) | 0.78 | (0.47-1.04) |
| SNRI | 3.1 | 5.80 | (4.01-8.34) | 0.78 | (0.70-0.86) |
| TCA | 42.8 | 3.44 | (2.51-4.77) | 0.86 | (0.82-0.90) |
| Trazodone | 1.6 | 2.91 | (0.77-6.36) | 0.90 | (0.73-1.01) |
| Bupropion | 1.3 | 3.98 | (0.22-9.45) | 0.88 | (0.70-1.03) |
| Mirtazapine | 0.0 | N/A^‡^ |  | N/A^‡^ |  |
| **Migraine** |  |  |  |  |  |
| SSRI | 0.0 | 73.26 | (0.00-200.54) | 0.60 | (0.00-1.01) |
| SNRI | 0.0 | 0.00 | (0.00-0.00) | 1.01 | (1.00-1.01) |
| TCA | 13.5 | 16.15 | (10.70-25.09) | 0.79 | (0.72-0.85) |
| Trazodone | 0.0 | N/A^‡^ |  | N/A^‡^ |  |
| Bupropion | 0.0 | N/A^‡^ |  | N/A^‡^ |  |
| Mirtazapine | 0.0 | N/A^‡^ |  | N/A^‡^ |  |
| **Fibromyalgia** |  |  |  |  |  |
| SSRI | 0.1 | 60.26 | (0.00-165.29) | 0.73 | (0.34-1.00) |
| SNRI | 3.1 | 52.52 | (30.34-98.60) | 0.74 | (0.62-0.84) |
| TCA | 3.4 | 13.56 | (7.58-21.76) | 0.74 | (0.60-0.87) |
| Trazodone | 0.0 | 50.10 | (0.00-162.60) | 0.51 | (0.00-1.02) |
| Bupropion | 0.3 | 150.51 | (68.67-415.60) | 0.30 | (0.00-0.50) |
| Mirtazapine | 0.0 | N/A^‡^ |  | N/A^‡^ |  |

Abbreviations: SSRI = selective serotonin reuptake inhibitor; SNRI = serotonin-norepinephrine reuptake inhibitor; TCA = tricyclic antidepressant

^†^SSRIs include citalopram, paroxetine, sertraline, escitalopram, fluoxetine, and fluvoxamine. SNRIs include venlafaxine, duloxetine, and desvenlafaxine. TCAs include amitriptyline, doxepin, trimipramine, nortriptyline, imipramine, clomipramine, and desipramine. Results are not shown for monoamine oxidase inhibitors, maprotiline, or nefazodone due to small numbers of prescriptions for each of these drugs.

^‡^Because no prescriptions for the antidepressant were written for the indication, the positive and negative likelihood ratio could not be calculated due to a zero denominator for the true positive rate (sensitivity) and false positive rate (1-sensitivity), respectively.

**APPENDIX C. (continued) Positive likelihood ratio (LR+) and negative likelihood ratio (LR-) of administrative diagnostic codes for the seven most common treatment indications, by antidepressant class**

| **Treatment indication, by antidepressant class^†^** | **Prevalence (%)** | **LR+ (95% CI)** | | **LR- (95% CI)** | |
| --- | --- | --- | --- | --- | --- |
| **Obsessive-compulsive disorder** |  |  |  |  |  |
| SSRI | 2.0 | 208.03 | (84.04-825.00) | 0.83 | (0.73-0.92) |
| SNRI | 0.7 | 83.54 | (0.00-466.86) | 0.94 | (0.81-1.00) |
| TCA | 0.4 | 1351.33 | (0.00-2083.32) | 0.84 | (0.55-1.00) |
| Trazodone | 0.0 | N/A^‡^ |  | N/A^‡^ |  |
| Bupropion | 0.1 | 0.00 | (0.00-0.00) | 1.00 | (1.00-1.00) |
| Mirtazapine | 0.0 | N/A^‡^ |  | N/A^‡^ |  |

Abbreviations: SSRI = selective serotonin reuptake inhibitor; SNRI = serotonin-norepinephrine reuptake inhibitor; TCA = tricyclic antidepressant

^†^SSRIs include citalopram, paroxetine, sertraline, escitalopram, fluoxetine, and fluvoxamine. SNRIs include venlafaxine, duloxetine, and desvenlafaxine. TCAs include amitriptyline, doxepin, trimipramine, nortriptyline, imipramine, clomipramine, and desipramine. Results are not shown for monoamine oxidase inhibitors, maprotiline, or nefazodone due to small numbers of prescriptions for each of these drugs.

^‡^Because no prescriptions for the antidepressant were written for the indication, the positive and negative likelihood ratio could not be calculated due to a zero denominator for the true positive rate (sensitivity) and false positive rate (1-sensitivity), respectively.
